# Acute Cholecystitis in a Gastric Bypass Patient Complicated by Takotsubo Cardiomyopathy

**DOI:** 10.1155/2022/5416092

**Published:** 2022-07-05

**Authors:** Muhammad Jawaid, Tarek El-Sherif, Aaron George, Hugo J. R. Bonatti

**Affiliations:** Meritus Health, Hagerstown, MD, USA

## Abstract

**Background:**

Gallbladder disease is a common condition after gastric bypass surgery. Even after weight loss, many bariatric patients continue to suffer from comorbid conditions. Takotsubo cardiomyopathy is a rare condition that mimics acute cardiac ischemia but seems to be caused by a catecholamine storm triggered by intense stress. *Case Report*. A 62-year-old female presented with acute right upper quadrant (RUQ) pain to the ER. She had a history of laparoscopic gastric bypass 5 years ago and had been noncompliant for 2 years. This noncompliance included missing follow-up appointments, gaining weight which caused poorly controlled DM, and not taking her vitamin supplements. Upon presentation, her WBC was elevated, her LFTs were normal, and imaging showed acute calculous cholecystitis. She was admitted and started on antibiotics with plans for laparoscopic cholecystectomy. The next day, she developed acute chest pain, and troponins were elevated with ST changes on EKG. Echocardiography showed a ballooned left ventricle indicative for Takotsubo cardiomyopathy. Symptomatic treatment including antibiotics, betablocker, and thiamine infusion was initiated. At three-month follow-up, ejection fraction had improved from <20% to >50%. The patient underwent interval laparoscopic cholecystectomy, which was technically very challenging due to severe ongoing acute and chronic cholecystitis. There were no cardiac issues, but the patient developed an abscess in the gallbladder fossa, which was successfully treated with oral antibiotics.

**Conclusions:**

Takotsubo cardiomyopathy complicating acute cholecystitis has thus far not been reported. Our patient had a history of gastric bypass and was noncompliant with vitamin supplementation. Thiamine deficiency may have contributed to the cardiac condition (wet beriberi).

## 1. Introduction

Bariatric surgery has been shown to be highly effective in achieving long-term weight loss. Compliance is one of the crucial factors in achieving sustained weight loss and preventing various complications. Even after adequate weight loss, many bariatric patients continue to suffer from comorbidities and patients require supplementation of vitamins, iron, and calcium. Thiamine deficiency may be an underestimated problem after weight loss surgery, and several cases of beriberi and Wernicke encephalopathy in these patients have been published (1–3). Morbidly obese individuals may be thiamine deficient prior to surgery. Further, studies suggest that patients after gastric bypass have a higher risk for postoperative thiamine deficiency than those having undergone sleeve gastrectomy or gastric banding (2, 4).

Gallbladder disease is a common condition following gastric bypass surgery, and some centers routinely give ursodeoxycholic acid after surgery to prevent formation of gallstones (5, 6). Nevertheless, biliary complications are reported in approximately 5% of bariatric patients with patients having had a gastric bypass having at much higher incidence. Traditionally, prophylactic cholecystectomy has not been recommended (5, 7).

Takotsubo cardiomyopathy (TC) is a rare condition that mimics acute cardiac ischemia and is attributed to a catecholamine storm triggered by intense stress including medical conditions (8–10). Primarily, ballooning of the left ventricle is the key finding on echocardiography and typically occurs in the apex (75-80%) and less commonly is found in the midventricle (10-20%) (9). A recent review by Kinno and Ono emphasized the importance of an interdisciplinary approach to TC due to the multitude of factors that may lead to the condition (10).

Cholecystitis-induced TC is a known phenomenon documented in at least two case reports (11, 12). Also, biliary pancreatitis has been complicated by TC, and TC may develop following laparoscopic cholecystectomy (13, 14). The development of TC is also documented following gastric bypass surgery (15).

We present a case of TC triggered by acute cholecystitis in a poorly compliant patient five years after Roux-En-Y gastric bypass surgery that may have been linked to thiamine deficiency.

## 2. Case Presentation

A 62-year-old female presented with acute right upper quadrant (RUQ) pain to the ER. The patient had a history of diabetes mellitus type 2, atrial fibrillation on apixaban, obesity, and laparoscopic gastric bypass surgery five years ago. The patient lost approximately 100 pounds within two years of surgery, and HbA1c reduced from 11.5 prior to surgery to 6.6 after surgery. However, the patient became noncompliant for the subsequent 2 years, causing her to regain greater than 40 pounds and a worsened glycemic index with HbA1c values ranging between 10 and 13.

**Figure 1:**
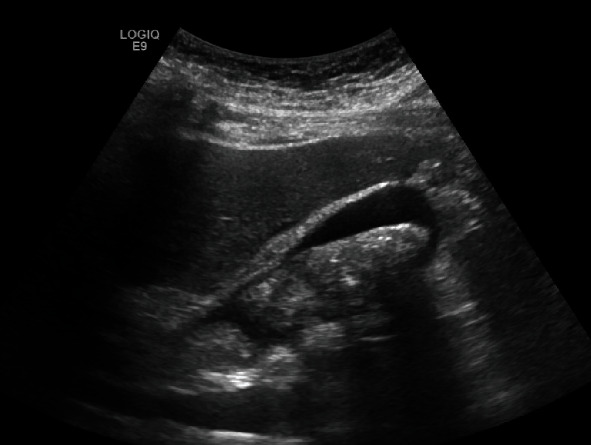
Abdominal ultrasound shows sludge in the gallbladder, gallbladder wall thickening, and pericolecystic fluid.

**Figure 2:**
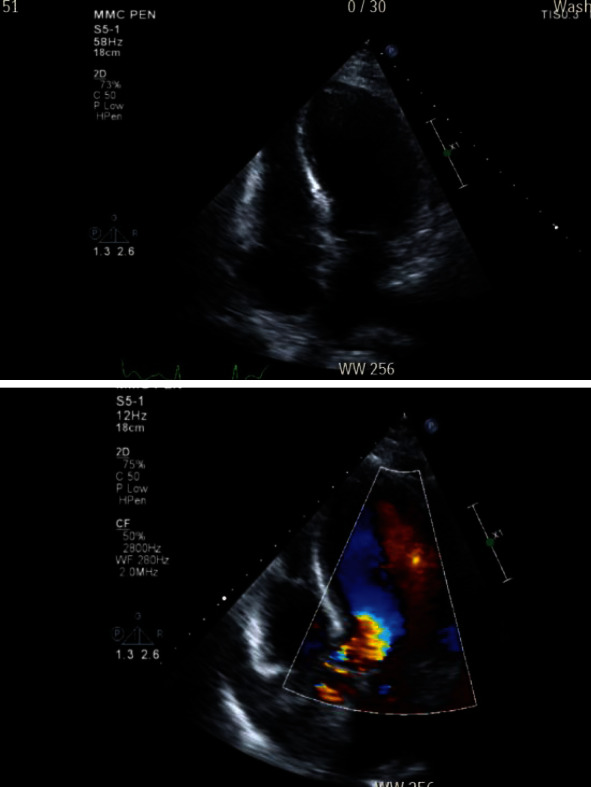
Echocardiography showing left ventricular ballooning.

**Figure 3:**
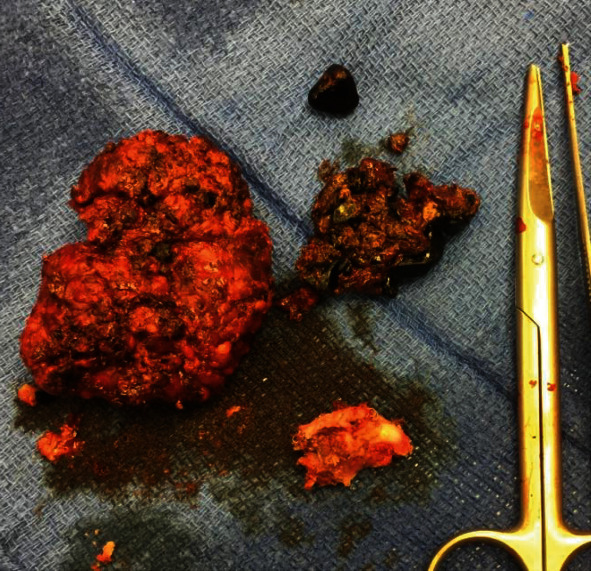
Gallbladder specimen: acute gangrenous and chronic cholecystitis.

The patient was also not taking her vitamin supplements. She reported general weakness and occasional shortness of breath and leg swelling. On admission, her WBC was elevated at 13.4 K/*μ*L, and CRP was 250, while total bilirubin and LFTs were normal. Abdominal ultrasound showed sludge and small stones within the gallbladder, as well as wall thickening and pericholecystic fluid consistent with acute calculous cholecystitis (Figure 1). She was admitted and started on antibiotics with plans for laparoscopic cholecystectomy during hospitalization. The following morning, the patient developed acute chest pain. Troponins were elevated to 5.42 ng/mL; lactic acid was 4.2 mmol/L. EKG demonstrated a right bundle branch block and premature ventricular complexes. Echocardiography showed severe hypokinesis of the mid- to distal anterior, anteroseptal, apical, and mid- to distal anterolateral walls which was consistent with TC (Figures 2(a)–2(b), video clip 1); ejection fraction was 20-25%. Symptomatic treatment including antibiotics for the acute cholecystitis and a beta-blocker for the TC were initiated. In addition, intravenous thiamine was given, but no thiamine levels were measured; she clinically improved over the next 24 hours. On repeat EKG, ST changes indicative for lateral ischemia had developed; BNP was 1192 pg/mL. Cardiac catheterization revealed mild nonobstructive coronary artery disease with 20-30% midleft anterior descending disease and severe LV dysfunction (video clip 2). The remaining coronary segments had no significant obstructive disease. Symptomatic treatment was continued; she was switched to oral antibiotics and after clinical improvement discharged for cardiac rehabilitation being deemed unfit for surgery. Intense counseling with regard to diet, weight loss and tight blood glucose control was done, and her thiamine levels were measured at low normal. At three-month follow-up, she had managed to lose 25 pounds and HbA1c came down to 7, while the ejection fraction had improved to >50%. She underwent interval laparoscopic cholecystectomy, which was technically very challenging due to severe ongoing acute and chronic cholecystitis (Figure 3). Pathology confirmed acute and chronic calculous cholecystitis with intramural abscesses and empyema. There were no cardiac issues, but the patient developed a fluid collection in the gallbladder fossa, which was successfully treated with oral antibiotics. She continued to lose weight and HbA1c maintained around 6.5. Oral thiamine supplementation was continued and thiamine levels maintained around 10.

A year later, she started to complain of epigastric pain, and endoscopy showed an anastomotic ulcer. Despite treatment with high dose protein pump inhibitors and sucralfate, the ulcer did not heal and she underwent successful laparoscopic revision of the gastrojejunostomy.

## 3. Discussion

To the best of our knowledge, this is the only case report of TC in the setting of cholecystitis after gastric bypass surgery. Aggarwal and Krantz described a TC case that occurred after cholecystitis and was resolved after cholecystectomy (11). Tori et al. reported TC developing after cholecystectomy for gallstones and common bile stones (12, 16, 17). Viegas et al. reported two cases of TC in the “early” postoperative period after bariatric surgery (15). We believe that thiamine deficient for being noncompliant with long-term postoperative care may have contributed to her cardiac condition, which entertains the possibility of patients having wet beriberi. Thiamine deficiency after bariatric surgery is very common and has been referred to as bariatric beriberi (1). TC has been observed in patients with thiamine deficiency in different settings such as alcohol abuse or anorexia nervosa and may be accompanied by Wernicke encephalopathy (18–20). Still, the exact mechanism of how thiamine deficiency could contribute to development of TC is unknown. During catecholamine storm, thiamine amongst other mechanisms may function in a protective way for the myocardium, and therefore, thiamine deficiency may increase the risk of TC in this setting (21).

TC is commonly caused by acute emotional stress, often among postmenopausal women, and involvement of psychiatry and/or neurology may be beneficial in the management (10). EKG changes such as ST or T wave changes in the anterior leads and elevated troponins should lead to suspicion of TC in certain patient populations especially if “stress” is reported and/or patients suffer from significant medical conditions. Diagnosis is made based on echocardiographic abnormalities including apical hypokinesis/ballooning (typical variant) as in our patient or midventricular hypokinesis (atypical variant) which are out of proportion to the degree of cardiac biomarker elevation. Coronary angiography is done in unclear cases and should demonstrate either normal vessels or nonobstructive atherosclerosis, but ventriculography will confirm diagnosis of TC such as in our case. Treatment of the underlying medical condition as in our patient—antibiotics for the acute cholecystitis and pain control—is crucial, and beta blockage remains the first-line therapy. In patients who are suspected to suffer from thiamine deficiency, supplementation seems to be indicated and this may be a focus of future studies in some TC patients.

In our patient, thiamine deficiency due to poor compliance following bariatric surgery possibly contributed to the development of TC in the setting of acute cholecystitis. The case emphasizes the importance of compliance after bariatric surgery to prevent significant complications as reported herein.

## Data Availability

Data cannot be made available due to HIPPA restriction.

## Supplementary Materials

Supplementary 1 Video clip 1: echocardiography—poor apical motion of the left ventricle (ballooning).

Supplementary 2 Video clip 2: ventriculography—severe dysfunction with poor apical contractility.
